# A systematic review and meta‐analysis of clinical predictors of lithium response in bipolar disorder

**DOI:** 10.1111/acps.13062

**Published:** 2019-06-30

**Authors:** T. P. Hui, A. Kandola, L. Shen, G. Lewis, D. P. J. Osborn, J. R. Geddes, J. F. Hayes

**Affiliations:** ^1^ Division of Psychiatry UCL London UK; ^2^ Department of Psychiatry University of Oxford Oxford UK

**Keywords:** bipolar disorder, clinical aspects, lithium

## Abstract

**Objective:**

To determine clinical predictors of lithium response in bipolar disorder.

**Methods:**

Systematic review of studies examining clinical predictors of lithium response was conducted. Meta‐analyses were performed when ≥2 studies examined the same potential predictor.

**Results:**

A total of 71 studies, including over 12 000 patients, identified six predictors of good response: mania‐depression‐interval sequence [odds ratio (OR): 4.27; 95% CI: 2.61, 6.97; *P* < 0.001], absence of rapid cycling (OR for rapid cycling: 0.30; 95% CI: 0.17, 0.53; *P* < 0.001), absence of psychotic symptoms (OR for psychotic symptoms: 0.52; 95% CI: 0.34, 0.79; *P* = 0.002), family history of bipolar disorder (OR: 1.61; 95% CI: 1.03, 2.52; *P* = 0.036), shorter prelithium illness duration [standardised mean difference (SMD): −0.26; 95% CI: −0.41, −0.12; *P* < 0.001] and later age of onset (SMD: 0.17; 95% CI: 0.02, 0.36; *P* = 0.029). Additionally, higher body mass index was associated with poor response in two studies (SMD: −0.61; 95% CI: −0.90, −0.32; *P* < 0.001). There was weak evidence for number of episodes prior to lithium treatment (SMD: −0.42; 95% CI: −0.84, −0.01; *P* = 0.046), number of hospitalisations before lithium (SMD: −0.40; 95% CI: −0.81, 0.01; *P* = 0.055) and family history of lithium response (OR: 10.28; 95% CI: 0.66, 161.26; *P* = 0.097).

**Conclusions:**

The relative importance of these clinical characteristics should be interpreted with caution because of potential biases and confounding.

**Summations**

- Our results suggest that predictors of good response are (i) mania‐depression‐interval sequence, (ii) absence of rapid cycling (iii) absence of psychotic symptoms, (iv) shorter prelithium illness duration, (v) family history of bipolar disorder and (vi) later illness onset. Additional features which may be related to response are body mass index, number of episodes before lithium treatment, number of hospitalisations before lithium and family history of lithium response.

**Limitations**

- Very few of the studies explored the possibility of interdependence or interaction between predictors.
- Because of the limitations of the data, particularly the limited number of RCTs, it is difficult to separate predictors of lithium response from predictors of a benign illness course.
- Because of the low reliability of the results and the inability to eliminate biases, any clinical conclusions relating to any single predictor should be made cautiously.

## Introduction

Globally, guidelines recommend lithium as first‐line maintenance treatment for bipolar disorder (BPD) 1, 2, 3. While lithium has a higher complete response rate than other mood stabiliser medication, only one in three patients will respond well to the drug 4. A number of studies have attempted to identify predictors of response from biological, genetic, clinical and psychosocial characteristics. A recent review of biomarkers to predict lithium response was somewhat discouraging 5. Genome‐wide association studies have developed a polygenic risk score for lithium response 6 and large biological marker studies are just beginning 7. However, despite enormous potential to improve our understanding of the lithium‐responding subtype of BPD, these approaches are unlikely to be able classify responders accurately without the inclusion of additional clinical features 6. We identified four reviews of multiple clinical lithium response markers, with the most recent attempt to meta‐analyses original studies published in 2005 8, 9, 10, 11. These reviews are limited in their scope as they are not systematic and fail to meet PRISMA standards 12. Factors associated with lithium response described in these reviews include the course of illness, family history of bipolar disorder, family history of lithium response, age at illness onset, number of bipolar hospitalisations, mania‐depression‐interval (MDI) course sequence, depression‐mania‐interval (DMI), continuous cycling (CC) (<4 episodes per year without euthymic intervals 13), rapid cycling (RC) (≥4 episodes per year 13) and bipolar II disorder (BPD II). We also identified reviews which examined single predictors: pretreatment episode count 14 and episode sequence 15. In the light of these issues, we systematically reviewed the existing literature on clinical predictors of lithium response in BPD and performed meta‐analysis where possible.

## Methods

This systematic review followed the MOOSE guidelines and PRISMA statement 12, 16.

### Eligibility criteria

We included randomised trial and observational studies, including adult participants diagnosed with BPD receiving lithium monotherapy. Studies that did not report separate analyses of patients treated with lithium were excluded. Studies examining the use of lithium for other indications (such as unipolar depression) were excluded. We considered studies to be eligible for inclusion if they reported an association between patient level factors (e.g. age at illness onset) and any definition of a lithium response (e.g. recurrence under lithium treatment).

### Information sources

We searched EMBASE, Medline and Web of Science from inception to July 2018; the final search was performed on July 14, 2018. Additional studies were identified through screening reference lists of included studies and relevant papers. We included only English language studies in humans. Other articles relevant to this topic were searched for via Google Scholar, using reference lists of relevant studies.

### Search

We used the following search terms to search all trials registers and databases: [Lithium* OR lithium blood level OR lithium carbonate OR lithium citrate OR treatment response* OR drug response* OR predictor*] AND [Bipolar disorder] AND [observational stud* OR controlled clinical trial* OR RCT OR randomised controlled trial*].

### Study selection

Eligibility screening was performed independently by three reviewers. The first author (TPH) screened the titles and abstracts of potential studies to determine inclusion, with a 20% random sample of records independently screened by two reviewers (AK and LS). Eligible studies were subsequently confirmed by the three reviewers (TPH, AK and LS) who independently checked the full text of all retrieved articles. Disagreement was resolved through discussion and consensus between TPH, AK, LS and JFH.

### Data collection process

One reviewer (TPH) extracted the following data from included studies and the second (LS) checked the extracted data, including author details, year of publication, types of study design, sample size, interventions investigated, comparison, outcome evaluation or definition of lithium response and key finding. Disagreements were resolved by discussion between TPH, AK, LS and JFH.

### Data items

Information was extracted from each included study on: (i) characteristics of study participants (including sample size and number of lithium responders and non‐responders; (ii) intervention details (dose, duration of lithium treatment); (iii) definition of a treatment response (number of recurrence under lithium treatment, reduction in time spent in hospital under lithium treatment, reduction of episode frequency, or improvement during lithium treatment based on valid scales, such as Illness severity index (ISI) 17, Affective Morbidity Index (AMI) 18 and ALDA scale 19); (vi) potential predictors examined; (v) summary results. Data sharing is not applicable to this article as no new data were created or analysed in this study.

### Risk of bias in individual studies

Three reviewers (TPH, AK and LS) independently rated each eligible study. The quality of each individual study was evaluated using the modified Downs and Black quality assessment scale (Table S1), which consists of 26 questions to evaluate both randomised and non‐randomised studies 20. Question 27 evaluating power was excluded as power should not be part of quality assessment as the aim of a meta‐analysis is to detect an effect from inconclusive or underpowered studies. Each criterion is worth one point, and a total score of 20 or above, between 15 and 19, and 14 or below is considered a study of good, fair and poor quality respectively. This quality assessment tool evaluates study reporting, external and internal validity including bias and confounding. Discrepancies between the two reviewers were resolved by discussion and consensus.

### Synthesis of results and risk of bias across studies

Meta‐analyses were performed after the four assumptions of homogeneity were assessed: (i) studies should be similar in terms of patients recruited; (ii) studies should be comparing the same intervention or exposure with similar controls, (iii) studies should be reporting the same outcomes, (iv) the effect of a predictor should ideally be in the same direction 21. Narrative analysis was carried out along with meta‐analysis if only some of the included studies met all of the criteria. For each meta‐analysis, where there were two or more studies using the same sample of patients, we excluded the smaller or earlier study.

Meta‐analysis using the DerSimonian and Laird random effect model was conducted for each predictor because we assumed heterogeneity existed across different studies, given the definitions of lithium response across studies were inconsistent 22. For binary outcomes, results of the primary studies were summarised as odds ratios (ORs). For continuous outcomes, results of the primary studies were summarised in standardised mean difference (SMD). Pooled ORs or SMDs and corresponding 95% confidence intervals were calculated if two or more studies reported the same clinical predictor.

A number of studies categorised patients with BPD as ‘partial responder’ in addition to ‘responder’ and ‘non‐responder’. In order to conduct the random‐effects pairwise analysis, we combined the group ‘partial responder’ and ‘non‐responder’ and formed the group ‘partial or non‐responder’ to avoid chances of data contamination that might impact the results of clinical predictors of lithium responders. Heterogeneity for each predictor was assessed using forest plots and a measure of inconsistency (*I* ^2^). Publication bias was examined visually through evaluating funnel plots. Stata version 15 was used for all analyses.

## Results

### Studies included

**Figure 1:**
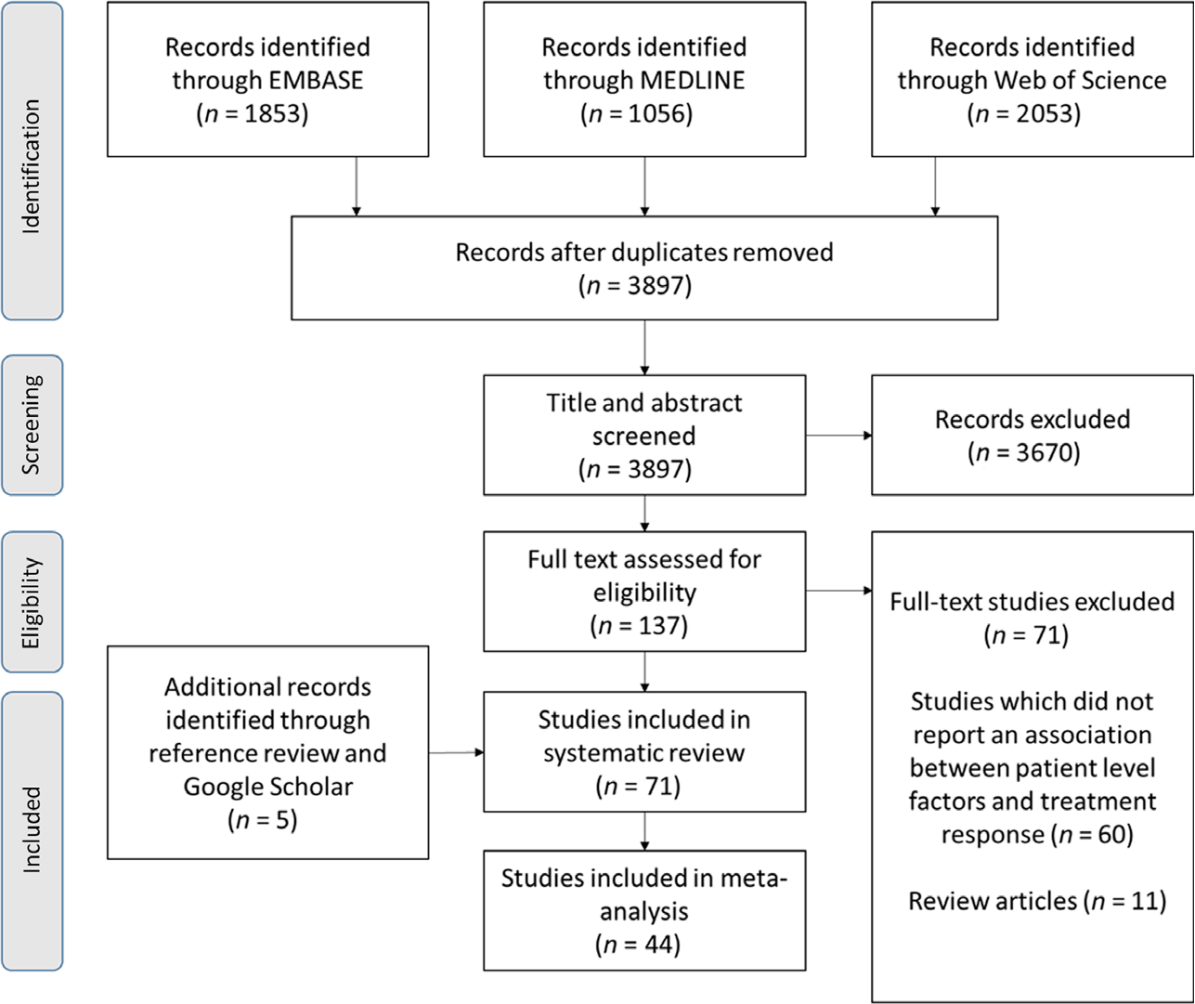
PRISMA flow diagram.

Our search resulted in 3897 unique citations. Of these, 3670 studies were excluded as the titles and abstracts were not relevant to the research topic, leaving 137 potentially eligible studies for which the full text was reviewed (Fig. 1). At this stage, 71 studies did not meet the inclusion criteria. An additional five studies that met the inclusion criteria were identified by checking the references of relevant papers and searching via Google Scholar. A total of 71 studies met all inclusion criteria and were included in systematic review, and 44 of these provided data which could be meta‐analysed. These studies are described in Table 1. Studies were excluded from the meta‐analysis if the population overlapped with another included study population or if it was not possible to calculate the OR or SMD. This meant two large studies using Danish population registers could not be included 23, 24.

**Table 1:** Characterisation of selected studies (*n* = 71) and the clinical predictors of lithium response

**Author**	**Year**	***n***	**Lithium level & treatment duration**	**Clinical Predictors (** * **included in meta‐analysis)**	**Definition of treatment response**	**Key Findings as reported in published study**	**Study quality**
Aronoff & Ebstein 55	1970	18	Level: na Duration: 3 years	Family history of affective disorder*	Recurrence under lithium	Five of seven patients with good response had a family history of affective disorder, compared to one of five in suboptimal responders (p‐value na)	POOR
Mendlewicz et al. 56	1973	43	Level: 0.8–1.3 mmol/l	Family history of affective illness Family history of BPD	Time to recurrence	Fifteen out of 24 responders to lithium had a positive family history of BPD, while only two out of the 12 non‐responders had a positive family history (0.02 < *P *< 0.05). Eighteen lithium responders had a positive family history of unipolar depression, compared with nine patients in the non‐responder group (p‐value NS)	
Prien et al. 57	1974	205	Level: 0.7 (mean) mmol/l, Duration: 2 years	Number of episodes prior lithium treatment Family history of BPD*	Reduction of episode frequency	55% patients in the low‐frequency group and 65% patients in the no‐episode group were lithium responders compared to 0% patients in the high‐frequency group (*P* < 0.01). The group with a family history of BPD had a higher proportion of responders (88%), than the group with no family history (49%), (*P*‐value NS)	FAIR
Dunner & Fieve 25	1974	55	Level: 0.7–1.2 mmol/l Duration: ≥6 months	Age at illness onset Sex Age at study start Subtype of BPD* RC*	Recurrence of mood episodes	Age at illness onset, age at study start, sex and type of BPD were not related to lithium response (*P*‐value na)	FAIR
Dunner et al. 58	1976	96	Level: na Duration: na	Sex* Number of episodes prior to lithium treatment Index episode polarity* cycling index episode Age at first treatment Age at illness onset Age at first hospitalisation Family history of affective illness* Family history of BPD*	Time to recurrence	Sex, number of previous episodes and age at first hospitalisation were associated with lithium response (*P* < 0.01) Nature of index episode, age at illness onset, age at first treatment and family history were not associated with lithium response (p‐value NS)	POOR
Ananth et al. 59	1979	59	Level: ≤0.8 mmol/l Duration: 2 years	Psychotic symptoms* Sex* Index episode polarity* Cyclothymic personality	Recurrence under lithium or need of co‐medication	Non‐responders (61%) displayed more psychotic symptoms during the duration of their illness (*P* < 0.05) Sex, type of index episode and cyclothymic personality were not a lithium response predictor (*P*‐value NS)	POOR
Rosenthal et al. 60	1979	66	Level: 0.5 to 1.2 mmol/l Duration: 9–15 months	Psychotic symptoms	Recurrence under lithium	Presentation of psychotic symptoms during mania appeared to be associated with good lithium maintenance response (*P* < 0.05)	FAIR
Kukopulos et al. 61	1980	294	Level: na Duration: na	MDI/DMI sequence* IRR* CC*	No recurrences for >1 year	MDI vs DMI odds ratio = 3.3 (*P* = 0.0002)	POOR
Sarantidis & Waters 62	1981	46	Level: na Duration: ≥2 years	Age at illness onset* Number of episodes/year prior to lithium treatment Family history of affective disorder* Sex*	Reduction in time spent in hospital under lithium treatment.	Age at illness onset, episode frequency, family history and sex were not associated with lithium response (*P*‐value na)	POOR
Abou‐Saleh 40	1983	53	Level: 0.6 to 1.3 mmol/l Duration: ≥2 years	Personality traits: extrovert; neuroticism, psychoticism; lie; dominance; intropunitiveness; extrapunitiveness	Recurrence under lithium	Deviant personalities were associated with poor lithium response. 24% responders were ‘deviant personalities’ and 57% non‐responders showed this abnormality (*P* < 0.05) The average score of neuroticism was significantly lower for lithium responders compared to non‐responders (*P* < 0.01)	POOR
Yang 63	1985	101	Level: 0.5–1.0 mmol/l Duration:>2 years	Age at illness onset Number of episodes prior lithium treatment	Reduction in episode frequency/admission	Age at illness onset and number of episodes prior lithium were not associated with lithium response (*P*‐value na)	POOR
O'Connell et al. 41	1985	60	Level: 0.5–1.2 mmol/l Duration: 92.4 months (mean)	Social support	Recurrence under lithium	High social support was associated with better functioning and fewer recurrent episodes (*P* < 0.05)	POOR
Abou‐Saleh & Coppen 64	1986	31	Level: 0.5–0.7 mmol/l Duration: 3 years	Sex* Number of previous episodes prior lithium treatment* Family history of BPD	Average morbidity index under lithium	Good responders had fewer episodes prior to lithium compared to fair to poor responders (*P* < 0.04) Patients with a family history of BPD had significantly higher lithium response than those with no family history (*P* < 0.05)	FAIR
Bouman et al. 65	1986	104	Level: na Duration: 10 years	Number of previous episodes prior to lithium treatment	Recurrence during lithium treatment	16% of BPD patients who were commenced on lithium treatment during their index episode relapsed compared to 32% BPD patients who had multiple episodes prior to lithium treatment (*P*‐value na)	FAIR
Mander 42	1986	98	Level: >0.4 mmol/l Duration: 3−6 months	Age at study start* MMRS (modified manic rating scale, severity rating) Duration of illness Sex* Social class Received treatment for previous episodes Extrovert/cyclothymic personality; Family history of affective disorder* Alcohol and drug use* Depressive symptoms Compulsory admission	Recurrence during lithium treatment	No significant differences between responder and non‐responders by any potential predictors	POOR
Goodnick et al. 66	1987	91	Level: 0.71 (±0.14) mmol/l Duration: 44.6 (±20.4) months	Sex Age at study start Subtype of BPD Age at illness onset RC Family history of any affective disorder Family history of BPD	Recurrence during lithium treatment	No significant differences between responder and non‐responders by any potential predictors	
Grof et al. 67	1987	50	Level: 0.7 mol/l (mean) Duration: ≥2 years	MDI/DMI sequence* IRR* CC*	Reduction of episode frequency	94% of MDI patients were lithium responders 55% of DMI patients were lithium responders 85% of IRR patients were lithium responders 33% of CC patients were lithium responder	POOR
Haag et al. 68	1987	93	Level: na Duration: na	MDI/DMI sequence* IRR* CC*	Reduction of number of hospitalisations per year	45% MDI patients were lithium responders 10% DMI patients were lithium responders 3% IRR patients were lithium responders 0% CC patients were lithium responders	POOR
Lusznat et al. 69	1988	54	Level: 0.6–4 mmol/l Duration: 6 weeks: double blind	Euphoric mood prior treatment Sex*	Recurrence during lithium treatment	Euphoric mood at admission was associated with poor lithium response but good carbamazepine response (p‐value na) 83% of poor lithium responders were male, compare to 20% of good responders (p‐value na)	GOOD
Faedda et al. 70	1989	40	Level: na Duration: na	MDI/DMI sequence* IRR* CC*	No recurrences for >1 year	MDI vs DMI odds ratio 2.7 (*P* = 0.31)	POOR
Maj et al. 71	1989	118	Level: 0.5–1.0 mmol/l Duration: na	MDI/DMI sequence* IRR* CC*	Average reduction in morbidity	MDI and IRR groups had a significant reduction of number of episodes after receiving lithium treatment compared to DMI and CC groups (*P* < 0.001) MDI and IRR groups had a significant reduction of total morbidity after receiving lithium treatment compared to DMI and CC groups (*P* < 0.001)	FAIR
Miller et al. 72	1991	53	Level: na Duration: na	Sex* Age at study start Number of hospitalisations prior to lithium* Psychotic symptoms Specific mood symptoms: elation, grandiosity, paranoia, agitation,irritability, aggressiveness	Global Assessment of Functioning	No difference between responders and non‐responders by included predictors	
O'Connell et al. 36	1991	248	Level: 0.5–1.0 mmol/l Duration: 8 years (±5.6)	Number of hospitalisations prior to lithium* Social support Social class Expressed family negative affective style Sex	Global Assessment Scale score under lithium	More frequent hospitalisations, lower social class and less social support was significantly associated with poorer outcome (*P* < 0.0001) Patients whose families showed high expressed emotion were over‐represented in the poorer outcome groups (*P* = 0.004) as were those whose families had a negative affective style (*P* < 0.0001)	FAIR
Okuma 30	1993	108	Level: n/a Duration: ≥2 years	Age at illness onset RC* CC* Predominant polarity* Type of BPD* Presentation of atypical symptomatology	Time ill during lithium treatment	Age at illness onset, presentation of atypical symptomatology and types of BPD were not associated with response (*P*‐value NS) RC was associated with a poor response (*P* < 0.01) Predominant mania over depression was associated with good lithium response (p‐value NS)	POOR
Gasperini et al. 26	1993	213	Level: 0.5–0.9 mmol/l Duration: na	Age at illness onset Duration of illness Age at study start Index episode polarity	Episode frequency during lithium treatment	Earlier onset of illness was associated with poor lithium response and higher recurrence (*P* < 0.0001) Longer duration of illness was associated with poor lithium response and higher recurrence (*P* < 0.02). Greater age receiving lithium treatment was associated with poor lithium response and higher recurrence (*P* < 0.04) Both manic and depressive index episodes were not associated with lithium response (*P*‐value NS)	FAIR
Grof et al. 73	1994	380	Level: ≥0.7 mmol/l Duration: 3–20 years	Family history of BPD* Family history of any affective disorder*	Recurrence during lithium treatment	A positive family history of BPD was associated with good lithium treatment response (*P*‐value na) BPD was more common in first‐degree relatives of patients who responded to lithium compared to those who did not respond (*P*‐value na)	FAIR
Maj et al. 74	1996	63	Level: 0.62 mmol/l (median) Duration: 5 years	Number of episodes before lithium treatment Number of previous hospitalisations Age at study start Prelithium illness duration Sex Age at first psychiatric contact Number of manic episodes before intake; Total morbidity in the 2 years preceding intake Family history of BPD	Recurrence during lithium treatment	Late non‐responders had more episodes of prior lithium treatment compared to stable responders (*P* < 0.05) Late non‐responders had more previous hospitalisations prior lithium treatment compared to stable responders (*P* < 0.01) Late non‐responders had an older mean age at receiving lithium treatment compared to stable responders (*P* < 0.05) Late non‐responders had a longer prelithium illness duration compared to stable responders (*P* < 0.02) Sex; age at first psychiatric contact; number of affective episodes before intake; manic episodes before intake; total morbidity in the 2 years preceding intake; and family history of BPD were not predictors of lithium response (*P*‐value NS)	FAIR
Denicoff et al. 75	1997	42	Level: 0.5–1.2 mmol/l Duration: 2 years	Age at study start* Age of first treatment Prelithium illness duration* Number of hospitalisations prior to lithium Depressive index episode	Morbidity during lithium treatment	Younger age at the time of study entry (*P* < 0.05), having a first treatment by age 20 or earlier (*P* < 0.01), short prelithium illness duration (*P* < 0.05), fewer hospitalisations (*P* < 0.05), and manifested a depressive index episode than manic index episode (*P* = 0.05) were associated with positive response to lithium	FAIR
Engstrom et al. 76	1997	98	Level: >0.4 mmol/l Duration: 10.0–13.0 years	Family history of any affective disorder Family history of unipolar depression Family history of BPD	Frequency of episodes per year	More episodes per year during lithium treatment were found in patients with a family history of first‐ or second‐degree relative with any affective disorder (*P* = 0.0119), and first‐ or second‐degree relative with BPD (*P* = 0.0012), when compared to non‐familial patients 88% of non‐familial patients had a reduction in episodes during lithium treatment, compared to 77% with a family history of any affective disorder, 84% with a family history of unipolar depression and 68% with a family history of BPD	
Greil et al. 77	1998	86	Level: 0.61 (±0.12) mmol/l Duration: 30 months	Classical features: without mood‐incongruent delusions, without comorbidity, and without mixed state	Time to hospitalisation	For the classical group (*N* = 67), lithium (*N* = 35) proved to be highly superior to carbamazepine in preventing hospitalisations (*P* = 0.005) but not for the non‐classical group (*P* = 0.075)	
Kusalic & Engelsmann 44	1998	29	Level: 0.80–1.30 mmol/l Duration: 2 years	Age at study start Prelithium illness duration Family history of BPD	Recurrence during lithium treatment:	Responders had an older mean age, longer duration of illness and presentation of family history of BPD compared to non‐responders (*P*‐value na)	FAIR
Maj et al. 37	1998	247	Level: 0.64 (±0.09) mmol/l Duration: 5 years	Number of hospitalisations prior to lithium* Number of episodes prior lithium treatment* Age at illness onset* Age at study start* Sex MDI/DMI sequence RC* Family history of BPD* Psychotic symptoms*	Number of recurrences during lithium treatment	Patients with fewer hospitalisations (*P* < 0.0001), fewer episodes before lithium treatment (*P* < 0.0001) and patients without a RC pattern (*P* < 0.00001) had better lithium response Age, age of first psychiatric contact, sex, DMI pattern, family history of BPD and psychotic features in index episode were not associated with lithium response (*P*‐value NS)	FAIR
Tondo et al. 38	1998	317	Level: na Duration: 6.35 years	Prelithium illness duration* Type of BPD* Sex Family history of affective disorder Education Marital status Employment status Age at onset RC Number of episodes prior to lithium	Time ill during lithium treatment	Prelithium illness duration was strongly negatively associated with clinical improvement (*P* < 0.0001) The proportion of lithium treatment‐responsive patient was significantly greater among BPD II subjects (65.1%) than BPD I subjects (51.1%) (*P* = 0.01). Other predictors were NS	FAIR
Franchini et al. 78	1999	179	Level: 0.5–0.9 mmol/l Duration: 48 months	Prelithium illness duration	Recurrence during lithium treatment	Beginning lithium treatment earlier predicted better outcome than beginning lithium treatment later (*P* < 0.00001)	FAIR
Kulhara et al. 45	1999	118	Level: 0.4–1.2 mmol/l duration: 11 years	Number of depressive episodes prior to lithium treatment Number of life events Total stress score Social support score Prelithium illness duration* Number of episodes prior to lithium treatment* Number of manic episodes prior lithium Number of hospitalisations prior to lithium* Polarity of index episode* Predominant polarity*	Reduction in episode frequency	Good responders had fewer depressive episodes prior lithium treatment (*P* < 0.05) and fewer of life events (*P* < 0.05) compared to partial/poor responders Good responders had lower total stress score compared to partial/poor responders (*P* < 0.05) Good responders had higher social support compared to partial/poor responders (*P* < 0.05) Illness duration prior lithium treatment; number of episodes prior lithium; numbers of manic episodes prior lithium; number of hospitalisation prior to lithium were not predictors of lithium response (*P*‐value NS)	FAIR
Yazici et al. 33	1999	141	Level: 0.75 (±0.08) Duration: ≥3 years	Age at illness onset Psychotic symptoms Episode severity Number of episodes prior to lithium Predominant polarity Number of hospitalisations prior lithium Type of BPD Ratio of psychotic episodes RC* CC* Family history of BPD Family history of any affective disorder	Affective morbidity index during lithium treatment	Greater age at disease onset (*P* < 0.002), mild and moderate episode severity (*P* < 0.0001), higher number of episodes prior lithium treatment (*P* < 0.00001), fewer hospitalisations prior lithium treatment (*P* = 0.007), BPD II (*P*‐value na) and low ratio of psychotic episodes (*P* < 0.00001) were associated with better lithium response Psychotic symptom (*P* < 0.00002) and mania predominance over depression (*P* < 0.00001) were associated with poor lithium response RC, CC, and family history of affective disorder and BPD were not predictors of lithium response (*P*‐value NS)	FAIR
Baldessarini et al. 79	2000	360	Level: 0.61 (±0.14) mmol/l Duration: 13.3 (±9.9) years	RC*	Recurrence during lithium treatment	Patients with RC were 13.7% less likely to be fully protected from all recurrences during lithium maintenance (*P* < 0.04)	FAIR
Coryell et al. 32	2000	186	Level: 0.72 mmol/l Duration: ≥26 weeks	Age at illness onset* Types of BPD Polarity of index episode* Age at study start* Sex* Alcohol and drug use*	Total morbidity score	Greater age at illness onset was associated with better lithium response (*P* = 0.0023) BPD I was associated with better lithium response compared to BPD II (*P* = 0.018) Pure mania index episode was associated with better lithium response (*P* = 0.003) Pure depression index episode (*P* = 0.048) and cycling were associated with poor lithium response (*P* = 0.020) Age, sex, alcohol and drug use were not predictors (*P*‐value NS)	FAIR
Schurhoff et al. 31	2000	97	Level: n/a Duration: ≥1 year	Age at illness onset	Recurrence during lithium treatment	64% of late onset (>40 years) patients responded to lithium compared to 43.3% of early onset (<18 years) patients (*P* = 0.04)	FAIR
Serretti et al. 27	2000	61	Level: 0.4–0.7 mmol/l Duration: 53 months	Prelithium illness duration Age at study start Age at illness onset Duration of illness Duration of lithium treatment	Recurrence during lithium treatment	Correlation between clinical variables and lithium response: Prelithium illness duration: *r* = −0.49 (*P* < 0.001) Age at study start: *r* = −0.13 (*P* = 0.312) Age at onset: *r* = 0.15 (*P* = 0.222) Duration of illness: *r* = −0.34 (*P* = 0.007) Duration of lithium treatment *r* = 0.31 (*P* = 0.015)	FAIR
Swann et al. 80	2000	35	Level: na Duration: 21 days	Number of episodes	Change in Schedule for Affective Disorders and Schizophrenia mania rating scores	Fewer manic episodes associated with better response (*P* = 0.01), no association with depressive episodes (*P* = 0.1)	GOOD
Tondo et al. 35	2001	360	Level: 0.61 (±0.14) mmol/l Duration: 6 (±5) years	Age at illness onset* Length of euthymic interval Number of episodes prior to lithium Prelithium illness duration*	Time ill during lithium treatment	Greater age at disease onset was associated with better lithium response (*P* = 0.005) Short interval between first and second episode was associated with better lithium response (*P* = 0.002) Higher number of episodes per year was associated with better lithium response (*P* = 0.006) Shorter prelithium illness duration was associated with better lithium response (*P* = 0.021)	FAIR
Viguera et al. 81	2001	360	Not reported but likely same as 35	Sex	Time ill during lithium treatment	No difference between sexes with respect to time ill. Time to relapse longer in women (*P* = 0.004)	
Grof et al. 19	2002	64	Level: n/a Duration: ≥1 year	Family history of lithium response*	Alda treatment response scale	67% of relatives of lithium responsive patients responded to lithium compared to 35% of the control group (*P* = 0.014)	FAIR
Swann et al. 82	2002	28	Level: na Duration: na	Psychotic symptoms Classic presentation	>50% improvement on Manic Syndrome Score	Individuals with psychotic symptoms and classical presentations responded equally well to lithium or valproate	
Hartong et al. 83	2003	44	Level: 0.75 mmol/l Duration: 2 years	Polarity of index episode Type of BPD	Recurrence during lithium treatment	Lithium was more effective than carbamazepine in patients with a (hypo)manic index episode, (*P* < 0.01) Lithium was more effective than carbamazepine in patients with BPD II (*P* < 0.05)	GOOD
Passmore et al. 84	2003	164	Level: na Duration: na	Pretreatment episodic illness course Family history of BPD	Recurrence of mood episode	Pretreatment episodic illness course was associated with good lithium response (*P* < 0.001) 16.6% of first‐degree relatives of lithium responders responded to lithium compared with 2.5% of such relatives of patients who responded to lamotrigine (*P* = 0.05)	FAIR
Washizuka et al. 85	2003	54	Level: 0.3–1.0 mmol/l Duration: 4.4 (±5.6) years	Sex* Age at illness onset* Age at study start* Types of BPD* Psychotic symptoms* RC* Family history of any affective disorder*	Recurrence during lithium treatment	Sex (*P* < 0.01), age at illness onset (*P* = 0.04) and RC (*P* = 0.04) predict response. All other covariates NS	
Bremer et al. 86	2007	184	Level: na Duration: na	PTSD comorbidity	Reduction in symptoms	People without PTSD had an improved lithium response rate compared with people with PTSD (*P* = 0.029)	POOR
Duffy et al. 87	2007	15	Level: ≥0.7 mmol/l Duration: ≥1 year	Family history of lithium response* Pretreatment episodic illness course	Alda treatment response scale	All patients who responded to lithium had lithium responsive parents (*P* = 0.001) 90% patients with episodic BPD responded better to lithium, and all patients with chronic BPD responded better to other mood stabilisers (*P* = 0.001)	FAIR
Garnham et al. 4	2007	78	Level: na Duration: ≥6 months	Age at illness onset* Type of BPD* Pretreatment episodic illness course	Alda treatment response scale	The full responders had earlier onset than non‐responders (*P* = 0.03) Full response to lithium was better in bipolar II disorder (*P* = 0.009) Full response to lithium was better in those with an episodic course of illness prior to treatment (*P* = 0.004)	FAIR
Rybakowski et al. 39	2007	111	Level: n/a Duration: ≥5 years	Age at illness onset* Age at study start* Prelithium illness duration* Number of episodes prior to lithium treatment* Duration of lithium treatment	Reduction in number of episodes	Age at illness onset, age at study start, prelithium illness duration, duration of lithium treatment and number of affective episodes before lithium treatment were not associated with lithium response (*P*‐values na)	POOR
Berghofer et al. 88	2008	242	Level: ≥0.5 mmol/l Duration: 10 (±6.4) years	Atypical symptoms	Total morbidity index (MI), depressive MI, manic MI	Atypical symptoms of BPD were not associated with lithium response (*P* = 0.472)	FAIR
Masui et al. 34	2008	161	Level: 0.4–1.2 mmol/l Duration: ≥1 year	Sex* Age at illness onset* Type of BPD*	Recurrence under lithium	Greater age at disease onset is associated with better lithium response (*P* < 0.01) 18% BPD II patients were responders, compared to 82% of BPD II non‐responders. 34.9% BPD I patients were responders, and 65.1% BPD I non‐responders. (*P* < 0.05). sex (*P*‐value NS)	POOR
Backlund et al. 28	2009	100	Level: 0.5 −0.9 mmol/l Duration: 9.7 (±5.8) years	Age at illness onset RC Mixed index episode Comorbidity	Number of mood episodes during lithium treatment	The absence of mixed episodes, rapid cycling, comorbidity or onset of illness at 20 years of age or later predicted a good response to lithium (*P*‐value na). Onset of illness after 20: RR 3.4 (*P* = 0.027) No comorbidity: RR 3.3 (*P* = 0.039) No mixed episodes before lithium: RR 3.5 (*P* = 0.025) No periods of rapid cycling before lithium: RR 7.3 (*P* = 0.025) High burden of mania before lithium: RR1.0 (*P* = 0.99) High burden of depression before lithium: RR 0.8 (*P* = 0.72)	POOR
Calkin et al. 48	2009	159	Level: na Duration: na	Body mass index*	Alda treatment response scale	Mean body mass index: complete responders < partial < non‐responders (*P* = 0.01)	
Pfennig et al. 89	2010	336	Level: na Duration: 10.5 (±7.0) years	Interepisode residual symptoms Mood‐incongruent psychotic symptoms RC Family history of psychiatric illness Number of episodes prior to lithium treatment	Recurrence under lithium	Recurrence rates increased in patients with interepisode residual symptoms (HR 1.45, 95% CI 1.15–1.83), mood‐incongruent psychotic features (HR 1.40, 95% CI 1.11–1.77) and RC (HR 1.86, 95% CI 1.11–3.14) and reduced in patients with family history (HR 0.67, 95% CI 0.49–0.92)	
Kessing et al. 23	2011	3762	Level: na Duration: 0.5–10 years	Sex Age at lithium start Employment status Polarity of index episode Number of hospitalisations prior to lithium Prelithium illness duration Drug use	Time to treatment failure	Increased rates of non‐response associated with being female (*P* = 0.002), being unemployed/retired compared to employed (*P* < 0.0001), depressive index episode (*P* < 0.0001), increased number of hospitalisations (*P* = 0.0002), prelithium illness duration (*P* = 0.02), drug use (*P* = 0.05)	
Degenhardt et al. 49	2012	230	Level: 0.6–1.2 mmol/l Duration: 52–72 weeks	Sex Polarity of index episode 11 item Young Mania Rating Scale item scores 21 item Hamilton Depression Rating Scale total score Age at illness onset Body mass index Number of episodes prior to lithium RC	Time to treatment failure	Onset age and body mass index uniquely predicted relapse in lithium vs. olanzapine‐ or valproate‐treated individuals. RC predicted relapse in lithium‐ and olanzapine‐treated individuals. Other covariates did not predict relapse	
Guloksuz et al. 90	2012	60	Level: 0.80 (±0.12) mmol/l Duration: na	Sex* Age at study start* Age at illness onset* Total episodes per year Number of hospitalisations prior to lithium* Body mass index*	Alda treatment response scale	Lithium responders had lower body mass index (SMD −0.65, 95% CI −1.23 to −0.08). No difference by sex, age at onset, age at study start or number of hospitalisations	
Martinsson et al. 91	2013	130	Level: 0.5–0.9 mmol/l Duration: na	Sex* RC* Type of BPD* Age at study start	Alda treatment response scale	RC and BPD II less common in lithium responders (*P* < 0.05). No difference by sex or age	
Rybakowski et al. 92	2013	71	Level: 0.5–0.8 mmol/l Duration: 15 (±8) years	Personality traits: hyperthymic; anxiety; cyclothymic; depressive; irritable	Alda treatment response scale	The response to lithium based on Alda scale correlated positively with hyperthymic temperament score (*r* = 0.31, *P* = 0.009), and negatively with anxiety (*r* = 0.27, *P* = 0.022), cyclothymic (*r* = 0.26, *P* = 0.032), and depressive (*r* = 0.23, *P* = 0.052) temperaments scores No correlation found between lithium response and irritable temperament (*r* = 0.020, *P*‐value NS)	FAIR
Tharoor et al. 93	2013	122	Level: ≥0.6 mmol/l Duration ≥2 years	Sex* Age at study start* Age at illness onset* Number of episodes prior to lithium treatment* Psychotic symptoms* Family history of BPD*	Recurrence under lithium	No difference in response by potential predictors	
Kessing et al. 24	2014	4714	Level: na Duration:0.5–16 years	Early (first contact) vs late initiation of lithium	Time to treatment failure	Early starters of lithium had reduced non‐response rates (*P* < 0.0001)	
Calkin et al. 47	2015	80	Level: na Duration: ≥6 months	Glucose metabolism	Alda treatment response scale	Insulin resistance or type 2 diabetes mellitus associated with poorer lithium response (*P* < 0.0001)	
Wei Shan et al. 43	2016	47	Level: 0.52–0.77 mmol/l Duration: 3 years	Sex* Ethnicity Family history of any affective disorder* Family history of BPD* Type of BPD* Psychotic symptoms* RC* Mixed episodes Predominant mood polarity* Polarity of index episode*	Proportion of time ill during lithium treatment	Predominance of depression over mania was associated with a good lithium response (*P* = 0.071) Sex; ethnicity; family history; type of BPD; psychotic Symptoms; RC; mixed episodes; predominance of mania over depression; and polarity of first mood episode were not predictors of lithium response (p‐value NS).	FAIR
Silva et al. 29	2016	40	Level: na Duration: ≥6 months	Sex Years of scholarship Age at onset Duration of the disorder Number of manic episodes Age at first manic episodes Number of depressive episodes History of suicide attempt Psychotic symptoms Number of mood episodes with psychotic symptoms History of tobacco use Family history of psychosis	Alda treatment response scale	High number of mood episodes with psychotic symptoms was associated with poor lithium response (*P* = 0.006), History or current tobacco use was associated with poor lithium response (*P* = 0.0048). Sex; years of scholarship; age at onset; duration of the disorder; number of manic episodes; age at first manic episodes; number of depressive episodes; history of suicide attempt; presence of psychotic symptoms; family history of psychosis were not related to lithium response.	FAIR
Etain et al. 50	2017	148	Level: na Duration: na	Type of BPD Polarity of index episode RC Mixed episodes Suicide attempt Alcohol use Cannabis use Panic disorder Social phobia GAD Physical abuse (childhood trauma questionnaire)	Alda treatment response scale	In multivariable analysis, poor response was associated with mixed episodes (*P* = 0.013) and physical abuse (0.005) In univariable analysis, poor response was additionally associated with alcohol use (*P* = 0.032) all other variables were NS.	
Saito et al. 94	2017	96	Level: na Duration: na	Sex* Age at illness onset* Age at study start* Family history of BPD* Psychotic symptoms* Number of episodes prior to lithium treatment* Years of education	Alda treatment response scale	Responders had fewer episodes prior to lithium (*P* = 0.012). sex (*P* = 0.379), age at study start (*P* = 0.993), education (*P* = 0.876), age at onset (*P* = 0.837), family history of BPD (*P* = 0.708) and psychotic features (*P* = 0.698) were not associated with response	
Scott et al. 53	2017	300	Level: na Duration: na	Sex Type of BPD Polarity of index episode Family history of BPD I Age at illness onset Duration of illness Prelithium illness duration RC Mixed episodes Alcohol and drug use Anxiety disorder Psychotic symptoms Number of mood stabilisers prior to lithium	Alda treatment response scale	In multivariable model, full response best predicted by age at illness onset (*P* = 0.015), prelithium illness duration (*P* = 0.028), family history of BPD I (*P* = 0.026) and alcohol and drug use (*P* = 0.033)	
Sportiche et al. 46	2017	300	Level: na Duration: 36 months	Sex* Age at study start* Age at illness onset* Prelithium illness duration* Type of BPD* Polarity of index episode* Seasonal pattern Psychotic symptoms* Mixed episodes RC* Attempted suicide Anxiety disorder Alcohol and drug use* Family history of BPD*	Alda treatment response scale	Mixed episode (*P* = 0.017) and alcohol use disorder (*P* = 0.015) were associated with poor lithium response Sex; current age; age at onset; duration of illness before lithium; BPD I; manic polarity at onset; seasonal pattern; psychotic symptoms; RC; attempted suicide; anxiety disorders; substance use disorders; family history BPD I and II were not lithium response predictors (*P*‐value NS)	FAIR

BPD, bipolar disorder; CC, continuous cycling; CI, confidence interval; DMI, depression‐mania‐interval sequence; HR, hazard ratio; IRR, irregular sequence; MDI, mania‐depression‐interval sequence; na, not available; NS, non‐significant (at *P* = 0.05); PTSD, posttraumatic stress disorder; RC, rapid cycling; RR, risk ratio.

* Data included in meta‐analysis.

In total, 19 clinical variables were identified from the articles and further assessed as predictors of lithium response in at least two or more studies: (i) age at study start, (ii) age at illness onset, (iii) prelithium illness duration, (iv) number of episodes prior lithium treatment, (v) number of hospitalisations prior to lithium, (vi) type of BPD (BPD I vs. BPD II), (vii) interval course sequence (MDI vs. DMI), (viii) CC, (ix) irregular sequence (IRR) (absence of any regular mania‐depression‐sequence), (x) RC, (xi) index episode (mania vs. depression), (xii) predominant polarity (mania vs depression), (xiii) family history of any affective disorder, (xiv) family history of BPD, (xv) family history of lithium response, (xvi) alcohol and drug use, (xvii) psychotic symptoms, (xviii) sex and (xix) body mass index (BMI).

### Age at illness onset

**Figure 2:**
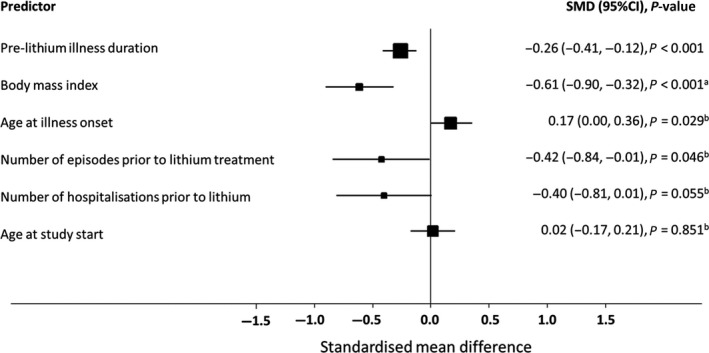
Relationship between clinical variables and lithium treatment response – standardised mean difference of continuous variables. ^a^only two studies; ^b^ *I* ^2^ > 50%.

A total of 21 studies explored the effect of age at illness onset; five studies 25, 26, 27, 28, 29 were excluded because of insufficient reporting; two studies reported categorical age data rather than continuous data and were therefore not included in meta‐analysis. The study by Okuma and colleagues 30 categorised patients into four age groups (>20; 21–30; 31–40; <40) and found no association between age at illness onset and lithium response. However, a similar study conducted by Schurhoff et al. 31 found that late onset (40 years old or older) was associated with good lithium response (*P* = 0.04). Pooling the remaining 14 eligible studies, with a total sample of 2063 patients, there was an association between age at onset and treatment response (SMD = 0.17; 95% CI: 0.02 to 0.33; *P* = 0.029; Fig. 2, Figure S1), but heterogeneity was high (*I* ^2^ = 58.3.6%; *P* = 0.003). Of these included studies, four found increasing age was associated with increased chance of lithium response 32, 33, 34, 35 and one found increased age was associated with a reduced chance of response 4.

### Age at study start

The association between age at study start and lithium treatment response was quantified in 10 studies with a total sample of 1266 patients. A medium level heterogeneity was observed (*I* ^2^ = 50.8%; *P* = 0.032). The pooled effect estimate suggested no association between study admission age and lithium response (SMD: 0.02; 95% CI: −0.17 to 0.21; *P* = 0.851; Fig. 2, Figure S1).

### Prelithium illness duration

Data from five studies with a sample of 931 patients were pooled (Table 1). Heterogeneity was low (*I* ^2^ = 0.0%; *P* = 0.701). The results suggested that a short prelithium treatment illness duration was associated with good lithium response (SMD = −0.26; 95% CI: −0.41 to −0.12; *P* < 0.001; Fig. 2, Figure S2). This was also true in the study by Kessing and colleagues of 4714 individuals with BPD 24; those commenced on lithium at first contact had lower rates of non‐response compared to those commenced at later contacts (HR 0.87, 95% CI 0.76 to 0.91, *P* < 0.0001).

### Number of episodes prior lithium treatment

The impact of mean number of episodes prior to lithium treatment on treatment response was assessed in seven studies with a total sample of 824 (Table 1). Meta‐analysis suggested that increased number of mood episodes prior to commencing lithium was weakly associated with reduced chance of good response (SMD = −0.42; 95% CI: −0.84 to −0.01; *P* = 0.046; Fig. 2, Figure S3). Heterogeneity was high (*I* ^2^ = 85.9%; *P* < 0.001).

### Number of hospitalisations prior to lithium treatment

A combined sample of 673 patients from four studies contributed data on number of previous hospitalisations. Although two studies suggested fewer hospitalisations were associated with good response 36, 37, overall there was no evidence of a clear association between number of hospitalisations and lithium response SMD = −0.40; 95% CI: −0.81 to 0.01; *P* = 0.055; Fig. 2, Figure S3). In the Danish population 23, increasing number of hospitalisations between diagnosis and starting lithium were associated with increased rates on non‐response (HR 1.03, 95% CI 1.02 to 1.05, *P* = 0.0002).

### Type of bipolar disorder

**Figure 3:**
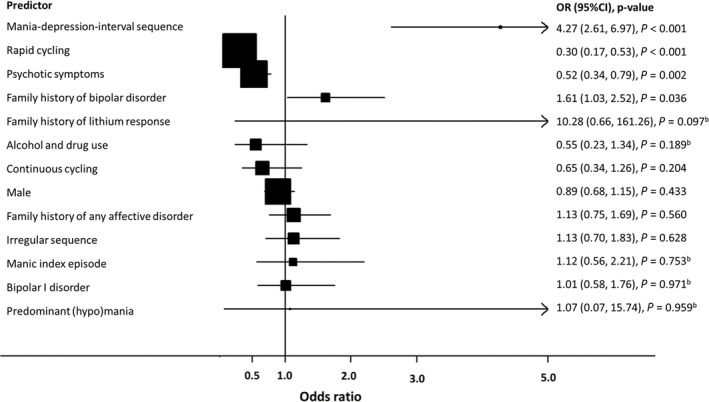
Relationship between clinical variables and lithium treatment response – odds ratios of binary variables. ^b^ *I* ^2^ > 50%.

The association between BPD subtype and good lithium response was quantified in 11 studies with a total of 1556 patients. There was evidence of considerable heterogeneity (*I* ^2^ = 70.7%; *P* < 0.001) across studies, and the result indicated insufficient evidence to support BPD I as a clinical predictor of lithium response when comparing to patients with BPD II (OR: 1.01; 95% CI: 0.58 to 1.76; *P* = 0.971; Fig. 3, Figure S4).

At an individual level, two of the included studies suggested BPD I may be associated with a preferential lithium response 32, 34 and three suggested BPD II may be associated with a preferential lithium response 4, 33, 38.

### Episode sequence

A total of six studies, including 340 patients, compared MDI and DMI sequence. MDI patients were more likely to be lithium responders than DMI patients (OR 4.27; 95% CI 2.61 to 6.97; *P* < 0.001; Fig. 3, Figure S5). Heterogeneity was low (*I* ^2^ = 0.0%; *P* = 0.680).

### Continuous cycling

The impact of continuous cycling on lithium treatment response was quantified in seven studies with a total of 804 patients. Meta‐analysis suggested no association between continuous cycling and response (OR: 0.65; 95% CI: 0.34 to 1.26; *P* = 0.204; Fig. 3, Figure S6).

### Irregular sequence

When the data from four studies of irregular sequence were pooled together, heterogeneity was low (*I* ^2^ = 0.0%; *P* = 0.496), and there was no association with lithium response (OR: 1.13; 95% CI: 0.70 to 1.83; *P* = 0.628, Fig. 3, Figure S6).

### Rapid cycling

The impact of the presence of RC on lithium treatment response was quantified in nine studies with a total of 1442 patients. Moderate heterogeneity was identified (*I* ^2^ = 37.5.6%; *P* = 0.119). The meta‐analysis result indicated evidence that patients displaying RC have reduced odds of lithium response compared to those without RC (OR: 0.30; 95% CI: 0.17 to 0.53; *P* < 0.001; Fig. 3, Figure S6).

### Polarity of index episode

There was no evidence of an association between lithium response and manic index episode (OR: 1.12; 95% CI: 0.56 to 2.21; *P* = 0.753; Fig. 3, Figure S7). From six studies, one suggested a manic index episode was a good predictor of response 32 and one suggested a depressive index episode was a good predictor 33. Others were inconclusive, and heterogeneity was high (*I* ^2^ = 73.7%; *P* = 0.002). Kessing et al. found reduced rates of non‐response in individuals with a manic index episode (HR 0.84, 95% CI 0.77 to 0.91) and elevated rates in those with a depressive index episode (HR 1.13, 95% CI 1.03 to 1.25) compared to those whose index episode was ‘remission, other or unspecified’. However, it is unclear who is included in this reference category and there is potential misclassification because of the routine register‐based nature of the data source.

### Predominant mood polarity

Predominant mania or depression was documented in three studies with a total sample of 280 patients. Overall, there was no evidence for an association between lithium response and mania over depression dominance (OR: 1.07; 95% CI: 0.07 to 15.74; *P* = 0.959; Fig. 3, Figure S8).

Included studies were contradictory; one study found a strong association between predominant mania and lithium non‐response (OR: 0.10; 95% CI: 0.04 to 0.25) 33, another found a strong association between predominant mania and lithium response (OR: 4.79; 95% CI 1.54 to 14.91) 30.

### Family history

Eight studies, including 714 individuals, contributed to meta‐analysis of the association between family history of any affective disorder and lithium response. There was no evidence of an association (OR: 1.13; 95% CI: 0.75 to 1.69; *P* = 0.560; Fig. 3, Figure S9). Individuals with a family history of bipolar disorder were more likely to have a good response to lithium (10 studies, 1454 patients**;** OR: 1.61; 95% CI: 1.03 to 2.52; *P* = 0.036; *I* ^2^ = 43.5%; heterogeneity *P* = 0.068; Fig. 3, Figure S9). One study, which could not be combined in meta‐analysis, runs contrary to this, finding 88% of individuals without a family history have a reduction in episode frequency during lithium treatment, while only 68% of those with a family history of BPD. Only two studies (79 patients) could be included in meta‐analysis of family history of lithium response. Both studies had point estimates suggesting good lithium response in family members may be associated with good response in the index patient, however, confidence intervals overlapped no effect (OR: 10.28; 95% CI: 0.66 to 161.26; *P* = 0.097, Fig. 3, Figure S9).

### Alcohol and drug use

The association between alcohol and drug use and lithium response was investigated in three studies with a total sample of 540 patients. The results showed a medium heterogeneity (*I* ^2^ = 54.5%; *P* = 0.111) and demonstrated no evidence to suggest alcohol and drug use as a potential predictor of lithium response (OR: 0.55; 95% CI: 0.23 to 1.34; *P* = 0.189; Fig. 3, Figure S10).

### Psychotic symptoms

A total sample of 1066 patients from eight studies were included in assessing psychotic symptoms. Medium heterogeneity was observed (*I* ^2^ = 42.8%; *P* = 0.093), and the result suggested a strong association between psychotic symptoms and poor response (OR: 0.52; 95% CI: 0.34 to 0.79; *P* = 0.002; Fig. 3, Figure S11).

### Sex

The role of sex as a potential lithium response predictor was investigated 1,729 patients from 17 studies. Sex was not associated with lithium treatment response (being male OR: 0.89; 95% CI: 0.68 to 1.15; *P* = 0.356; *I* ^2^ = 22.7%; heterogeneity *P* = 0.191; Fig. 3, Figure S12). However, the only population‐based study identified suggested an association between being female and non‐response (HR 1.12, 95% CI 1.04 to 1.21, *P* = 0.002) 23.

### Body mass index

BMI was investigated as a predictor in only two studies including 336 patients. In both studies, lower BMI was associated with better lithium response (pooled SMD: −0.61; 95% CI: −0.90 to −0.32; *P* < 0.001; *I* ^2^ = 0.0%; heterogeneity *P* = 0.111 Fig. 2, Figure S13).

### Further potential predictors

A study by Rybakowski et al. investigated the relationship between temperament and lithium response 39. Data from 71 patients suggested that lithium response was correlated positively with hyperthymic score (*r* = 0.31; *P* = 0.009), and negatively with anxiety and cyclothymic temperament scores (*r* = −0.27; *P* = 0.022 and *r *= −0.26; *P* = 0.032 respectively). We identified one other study which examined personality traits and treatment response 40. This study reported that responders had higher dominance scores (*P*‐value < 0.05), lower neuroticism scores (*P*‐value < 0.01) and were less likely to have ‘deviant personalities’ (*P*‐value < 0.05). Social support was examined in two studies with overlapping study populations 36, 41 and a third study which presented results in a way that did not permit meta‐analysis. Lower social support was associated with poor response in each case. Other sociodemographic characteristics were reported in a small number of studies. Social class was associated with response in one identified study, but not in another 36, 42. Education, marital status, 38 and ethnicity 43 were not associated with lithium response. Employment status was associated with response in one large nationwide population study 23, but not in a smaller observational study 38. Insulin resistance was found to be associated with poor response to lithium in one study, in keeping with the studies showing an association with BMI 47, 48, 49. While we did not consider childhood trauma as a ‘clinical’ predictor of treatment response, one included study examined this among other features 50. This study suggested physical abuse was an independent predictor of poor lithium response after accounting for many clinical characteristics. However, the only other study we could identify examining childhood trauma found no association between lithium response and any type of trauma 51.

### Risk of bias within studies

Overall, the mean Downs and Black quality assessment score was 16.3, which is considered fair quality. We identified eight good quality studies, 45 fair quality studies and 18 poor quality studies (Tables S1 and S2). Most of the studies failed to report or account for appropriate confounders in regression analyses.

### Risk of bias across studies

In line with the Sterne et al. 52, funnel plot asymmetry was assessed when 10 or more studies were included in the meta‐analysis. Funnel plots were produced for age at illness onset (Figure S14), sex (Figure S15), family history of BPD (Figure S16), age at study start (Figure S17) and type of BPD (Figure S18). The studies of BPD subtype, sex and family history produced asymmetrical funnel plots. A possible source of this asymmetry is true heterogeneity between studies; potentially because of differences in lithium dosage, treatment duration or diagnostic definition, small sample sizes and the low number of studies included.

## Discussion

We identified a total of 71 studies, including over 12 000 patients which explore clinical predictors of lithium treatment response in patients with BPD. From these, six predictors of good response were identified. Our results suggest that predictors of good response are (i) MDI sequence, (ii) absence of RC, (iii) absence of psychotic symptoms, (iv) shorter prelithium illness duration, (v) family history of bipolar disorder and (vi) later illness onset. Additional features which may be related to response are body mass index, number of episodes before lithium treatment, number of hospitalisations before lithium and family history of lithium response.

Our findings generally correspond with previous review articles 8, 9, 10, 11. As far as we are aware, Kleindienst et al. conducted the only previous meta‐analysis of multiple clinical response predictors and our results were broadly similar 8. However, we did not find a strong association with number of previous hospitalisations or CC, and they found no association with prelithium illness duration, psychotic symptoms or RC. This may be because of differing approaches to study inclusion and analysis, and in some cases because contradictory results have been found in individual studies published since 2005. Additionally, prelithium illness duration, number of episodes prior to lithium treatment and number of hosptialisations prior to lithium are likely to all be measuring a similar underlying concept.

Clinically, these predictors are likely to be of varying importance. Some may essentially reflect establishing a more benign illness course because of early intervention and may not be specific to lithium. This may be the case for shorter prelithium illness duration, and fewer episodes prior to lithium treatment, which are clearly related to illness severity. Others may be more central to guiding the choice to use lithium. DMI sequence, rapid cycling and psychotic symptoms are all associated with poor lithium response, so their presence may suggest an alternative treatment might be more appropriate for the patient. However, there is limited evidence to suggest any other drug therapy would lead to better than responses than with lithium. Family history of bipolar disorder and potentially family history of lithium response (likely under powered in our analysis) are important as they may reflect a more heritable subtype of BPD.

### Limitations

The reliability of the potential predictors identified remains unclear. For most of the meta‐analyses conducted, estimates were highly heterogeneous, often including studies suggesting both a positive and negative effect of the predictor. Most studies were rated as fair or poor in terms of quality. Often insufficient statistical information was reported in the primary study to conduct meta‐analysis; most studies failed to report adequate summary statistics such as standard deviation or number of responders and non‐responders. Sample sizes were often small and studies consisted of highly selective groups of patients. Also, the definition of lithium response in many of the studies did not rely on a standardised tool, which can greatly influence the process of identifying lithium responders and lithium non‐responders. As shown in Table 1, most of the studies relied on recurrence of an affective episode under lithium treatment to define lithium non‐responders. However, this definition of lithium response fails to consider changes in episode frequency or symptom severity, and so may miscategorise responders and non‐responders. Scott and colleagues note that using continuous scores for lithium response as opposed to categories of response leads to different predictors being identified 53. Additionally, none of the studies reported lithium plasma level or adherence to treatment by response status. Information on these factors would strengthen the argument that these are true predictors of lithium response as it would then be possible to rule out differences in the way treatment is used as a cause of the observed associations.

Very few of the studies explored the possibility of interdependence or interaction between predictors. For example, interdependence might exist between prelithium illness duration and illness severity 54. A greater illness severity is related to receiving early treatment and subsequently decreasing illness morbidity. Accordingly, a short prelithium illness duration might appear to be related to good lithium response 54. Only some of the more recent studies included multiple covariates in the same model (for example; 23, 50, 53) an approach which is necessary to determine whether covariates are truly *independent* predictors.

Because of the low reliability of the results and the inability to eliminate biases, any clinical conclusions relating to any single predictor should be made cautiously. Because of the limitations of the data, particularly the limited number of RCTs, it is difficult to separate predictors of lithium response from predictors of a benign illness course.

In conclude although we identified six potential clinical predictors of lithium response, there are a number of issues relating to their reliability and validity which cannot be addressed by reviewing the existing literature. As with response classification by genetic or biological markers, clinical response prediction is likely to be complex and multivariable. Studies need to explore multiple predictors, and their interactions, with operationalised end points for lithium response.

## Funding

AK is supported by the Economic and Social Research Council (ES/P000592/1). GL, DPJO and JFH are supported by the National Institute for Health Research UCLH Biomedical Research Centre. JRG is a National Institute for Health Research Senior Investigator. JFH is a Wellcome Trust Clinical Research Career Development Fellow (211085/Z/18/Z).

## Declaration of interest

None.

## Supporting information


**Table S1.** Modified Downs and Black checklist for the assessment of methodological quality of both randomized and non‐randomized studies.
**Table S2** **.** Study quality scores using modified Downs and Black scale: Checklist for measuring study quality (*n* = 50).
**Figure S1**. Relationship between age at illness onset, age at study start and lithium treatment response.
**Figure S2**. Relationship between pre‐lithium illness duration and lithium treatment response.
**Figure S3**. Relationship between number of episodes, number of hospitalizations prior lithium treatment and lithium treatment response.
**Figure S4**. Relationship between bipolar I disorder, bipolar II disorder and lithium treatment response.
**Figure S5**. Relationship between types of episodic sequence and lithium treatment response.
**Figure S6**. Relationship between types of cycling and lithium treatment response.
**Figure S7**. Relationship between depressive index episode, manic index episode and lithium treatment response.
**Figure S8**. Relationship between predominant mood polarity and lithium treatment response.
**Figure S9**. Relationship between types of family history and lithium treatment response.
**Figure S10**. Relationship between alcohol and drug use and lithium treatment response.
**Figure S11**. Relationship between psychotic symptoms and lithium treatment response.
**Figure S12**. Relationship between sex and lithium treatment response.
**Figure S13**. Relationship between Body mass index and lithium treatment response.
**Figure S14**. Funnel plot of studies examining age at illness onset as a predictor of lithium response, with pseudo 95% confidence limits.
**Figure S15**. Funnel plot of studies examining sex as a predictor of lithium response, with pseudo 95% confidence limits.
**Figure S16**. Funnel plot of studies examining family history of bipolar disorder as a predictor of lithium response, with pseudo 95% confidence limits.
**Figure S17**. Funnel plot of studies examining age at study start as a predictor of lithium response, with pseudo 95% confidence limits.
**Figure S18**. Funnel plot of studies examining bipolar disorder subtype as a predictor of lithium response, with pseudo 95% confidence limits.
